# Effect of Combined Live Probiotics Alleviating the Gastrointestinal Symptoms of Functional Bowel Disorders

**DOI:** 10.1155/2020/4181748

**Published:** 2020-09-17

**Authors:** Jin Shi, Feng Gao, Jie Zhang

**Affiliations:** Digestive Department, Beijing Anzhen Hospital, Capital Medical University, Beijing 100029, China

## Abstract

**Objective:**

Changes of the gut microbiota are related to the pathogenesis of functional bowel disorders (FBDs), and probiotic supplementation may be an effective treatment option. Therefore, we aimed to investigate the effect of combined live probiotics on the gastrointestinal symptoms of FBDs via altering the gut microbiota.

**Methods:**

Patients with the gastrointestinal symptoms of FBDs attending the Outpatient Department, from July to November 2019, were recruited. After the bowel preparation with polyethylene glycol electrolyte powder and colonoscopy, patients with normal result of colonoscopy were randomly divided into the probiotics group and control group. Patients in the probiotics group were prescribed with combined live *Bacillus subtilis* and *Enterococcus faecium* enteric-coated capsules for 4 weeks. Small intestinal bacteria overgrowth (SIBO) was measured by lactulose hydrogen breath test, and the microbial DNA was extracted from the fecal samples and the bacteria were classified by 16S rDNA gene amplicon sequencing.

**Results:**

Twenty-five patients of each group were recruited, and there was no significant difference between the probiotics and control groups on baseline gastrointestinal symptom rating scale (GSRS), positive rate of SIBO, and relative abundances of the gut microbiota at the phylum level. After 4 weeks of treatment, the values of the probiotics and control groups were as follows: GSRS 1.4 ± 1.4 and 3.6 ± 1.6 and positive rate of SIBO 28.0% and 56.0%, respectively. The median relative abundances of the gut microbiota were 1.01% and 5.03% Actinobacteria and 43.80% and 35.17% Bacteroidetes at the phylum level; 0.76% and 3.29% *Bifidobacterium*, 0.13% and 0.89% *Cillinsella*, 0.03% and 0.01% *Enterococcus*, 0.18% and 0.36% *Lachnospiraceae*, 0.10% and 0.16% *Ruminococcus torques group*, 1.31% and 2.44% *Blautia*, and 0.83% and 2.02% *Fusicatenibacter* at the genus level (*P* < 0.05), respectively.

**Conclusion:**

Combined live probiotic supplementation after the bowel preparation can alter the gut microbiota, decontaminate SIBO, and alleviate the gastrointestinal symptoms of FBDs. This trial is registered with ChiCTR1900026472.

## 1. Introduction

Gut flora plays an important role in human health and is closely related to the pathogenesis of many chronic diseases, especially benign and malignant disorders of the gut [1–3]. Functional bowel disorders (FBDs) are functional middle or lower gastrointestinal disorders with predominant symptoms or signs of abdominal pain, abdominal bloating, and bowel habit abnormalities (constipation, diarrhea, or mixed constipation and diarrhea); recent evidence suggest that changes in the gut microbiota are related to the pathogenesis of FBDs, and a combination of specific probiotics and synbiotics may be an effective treatment option for FBDs [4, 5].

Our clinical experience found that many gastrointestinal symptoms of FBDs can be alleviated after the bowel preparation, colonoscopy, and following treatment of combined live *Bacillus subtilis* and *Enterococcus faecium*, but lack proof of randomized controlled studies. Some studies showed that bowel preparation can alter the composition of the gut microbiota [6, 7], and probiotic supplementation is effective in alleviating patients' irritable bowel syndrome (IBS) [8], but no study reports about the effect of the bowel preparation and the following probiotics treatment on the gastrointestinal symptoms and gut microbiota of FBDs. Therefore, we propose the hypothesis that bowel preparation and following probiotic supplementation may alter the composition of the gut microbiota and alleviate the gastrointestinal symptoms of FBDs, and the aim of this study was to prospectively randomized controlled investigate the effect of combined live probiotics on the gastrointestinal symptoms and gut microbiota of FBDs after the bowel preparation and colonoscopy.

## 2. Materials and Methods

### 2.1. Subject Selection

Patients diagnosed with FBDs with the gastrointestinal symptoms of abdominal pain, abdominal bloating, abdominal distension, or bowel habit abnormalities (constipation, diarrhea, or mixed constipation and diarrhea) were enrolled prospectively from July 2019 to November 2019.

The inclusion criteria included the following: (1)25-70 years of age; (2) repeated episodes of abdominal pain, abdominal distension, or bowel habit abnormalities (constipation, diarrhea, or mixed constipation and diarrhea), with a period of more than 6 months; (3) no past history of any chronic disease; and (4) no history of abdominal surgery.

The exclusion criteria included the following: (1) contraindications for colonoscopy, disable to tolerate colonoscopy and bowel preparation; (2) taking drugs affecting the gut microbiota within a month before the selection (e.g., antibiotics, antiacid drugs, and probiotics) and drinking alcohol; (3) colonoscopy results of colon malignant or benign tumor, colorectal enteritis, colorectal ulcers, and inflammatory bowel disease; and (4) pregnant women.

Withdrawal criteria included the following: (1) poor compliance, (2)self-exit, and (3)taking drugs affecting the gut microbiota during the study (e.g., antibiotics, antiacid drugs, probiotics) and drinking alcohol.

### 2.2. Ethics

Our research was approved by the Ethics Board of Beijing Anzhen Hospital (approval no. 2019015). And all subjects signed informed consents.

### 2.3. Data Collection

Gastrointestinal symptom rating scale (GSRS) covers 15 gastrointestinal symptoms, each classified into four severity categories (score of 0–3) [9]. The GSRS scores and weight of all subjects were registered at baseline (before the colonoscopy) and 2 weeks and 4 weeks after the colonoscopy.

### 2.4. Lactulose Hydrogen Breath Test and Colonoscopy

Patients undertook lactulose hydrogen breath test to diagnose small intestinal bacteria overgrowth (SIBO) before and 4 weeks after colonoscopy and treatment [10]. The patients first exhaled baseline fasting breath samples, drank lactulose 10 g in 250 ml clear water, followed with subsequent breath samples acquired in 15-minute intervals for the period of 120 minutes. Breath samples were tested with Quintron gas chromatography. Based on previously published literature, a positive LHBT or SIBO was defined by one of the following criteria: (1) ≥20 ppm rise in hydrogen value within the first 90 minutes of lactulose administration compared with the baseline hydrogen value or (2) baseline hydrogen value ≥ 20 ppm and hydrogen value of 30 minutes after lactulose administration ≥20 ppm. In addition, avoid spicy foods, garlic, fruits, vegetables, grains, beans, bran cereals, drinking alcohol and smoking 24 hours before the test, and fasting for 12 hours before the test.

Bowel preparation of colonoscopy involved the consumption of four boxes of polyethylene glycol electrolyte powder (PEG; Staidson Beijing Biopharmaceutical Co., Ltd.) with 3.0 L clear water on the morning of the examination. And all patients undertook afternoon colonoscopy by specified gastroenterologists with a same-day bowel preparation.

### 2.5. Fecal Sample Collection

Fresh fecal samples (2 to 5 g) were immediately placed into a sterile sampling box, transferred by ice bath, and maintained at −80°C until use, and the first fecal sample of each patient was collected before bowel preparation [11].

### 2.6. DNA Extraction, Sequencing, and Bioinformatic Analyses

Microbial DNA extraction, amplification and sequencing, and bioinformatic analyses were followed by the methods of Fontana et al. [12].

The fecal DNA of each sample was extracted and purified from 300 mg feces using the StoolGen DNA Kit (CW2092, Beijing Cowin Bioscience Co., Ltd.), according to the manufacturer's instructions. The concentration and purity of the genomic DNA were measured with an agarose gel electrophoresis to determine DNA samples without degradation.

The gene-specific sequences used in this research target the 16S V3 and V4 regions. The 16S rDNA metagenomic sequencing libraries were prepared following the instructions (Illumina, Inc., San Diego, CA, USA). Each 16S library was sequenced in a separate 250 bp, paired-end run on the platform of Illumina HiSeq2500. Fast quality filter in the FASTX Toolkit 0.0.14 filtered the low-quality reads, and USEARCH 64 bit v8.0.1517 removed chimera reads. Operational taxonomic units (OTU) were aligned using the UCLUST algorithm and taxonomically classified using the SILVA 16S rRNA database v128. Alpha and beta diversities were generated by Quantitative Insights Into Microbial Ecology (QIIME) and calculated based on weighted and unweighted UniFrac distance matrices. The sequencing and bioinformatic analyses were performed by The Institute of Microbiology, Chinese Academy of Sciences (Beijing, China).

The Firmicutes to Bacteroidetes ratio was calculated as the ratio of the relative abundance of Firmicutes to Bacteroidetes, and Actinobacteria to Bacteroidetes ratio was calculated as the ratio of the relative abundance of Actinobacteria to Bacteroidetes.

### 2.7. Study Groups and Blinding

Patients were randomly divided into the probiotics group and control group by random number table, if the result of colonoscopy was normal. And patients in the probiotics group were treated with Medilac-S (live combined *Bacillus subtilis* and *Enterococcus faecium* enteric-coated capsules, 500 mg per time, three times per day, Hanmi Pharm Co. Ltd., Beijing, China) for 4 weeks.

Dr. Shi, Dr. Gao, and the patients understood the grouping information. Nurses and microbiological inspectors, who are in charge of performing breath test and the gut microbiota and collecting values of weight and GSRS, did not know the grouping information.

### 2.8. Statistical Analyses

Data were presented as numbers and proportion, mean and standard deviation (SD), median, and interquartile range (IQR) where appropriate and were compared using independent sample *t*-test, paired sample *t*-test, chi-square test, Mann–Whitney test, and Wilcoxon test where appropriate. Linear regression analysis was performed to investigate the influence of probiotics treatment, age, gender, baseline BMI, and obesity on the changes of the gut microbiota, GSRS, and SIBO between 4 weeks after colonoscopy and treatment and baseline. All tests were two-tailed and *P* values < 0.05 were considered significant. Statistical analyses were performed using the statistical software package SPSS for Windows, version 21 (SPSS, Chicago, IL).

### 2.9. Sample Size Calculation

The main index of the study was to evaluate the changes of GSRS. Group sample sizes of 17 can achieve 81% power to detect an estimated difference of 1.0 between the probiotics group 2.0 ± 1.0 and the control group 1.0 ± 1.0 with a significance level (alpha) of 0.05 using a two-sided two-sample *t*-test calculated by PASS 11 software. We did 25 cases in each group that were able to fully meet the need to detect the difference.

## 3. Results

### 3.1. Participant Characteristics and Clinical Data

**Figure 1:**
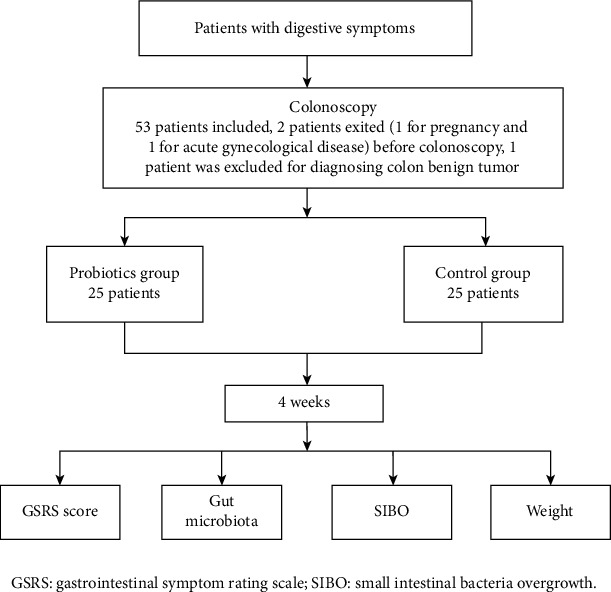
Flow chart of enrollment.

**Figure 2:**
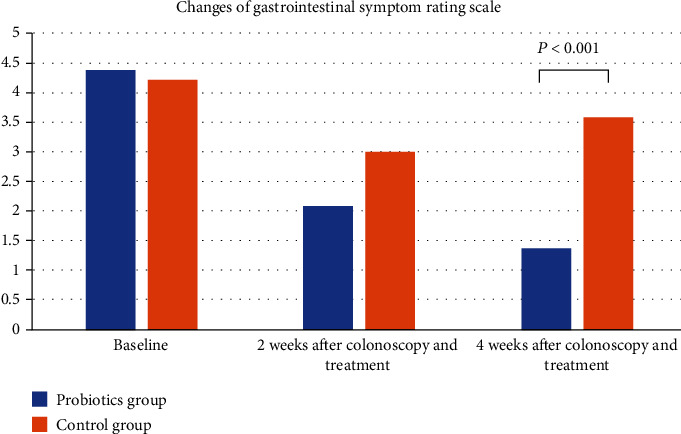
Changes of gastrointestinal symptom rating scale (GSRS). GSRS scores at the time of 2 weeks and 4 weeks after colonoscopy and treatment were significantly lower than those at the baseline of the probiotics and control groups. But GSRS score at the time of 4 weeks after colonoscopy and treatment was significantly higher than that at the time of 2 weeks after colonoscopy and treatment in the control group.

The flow chart was showed in Figure 1. 25 patients (male/female 9/16, age 40.6 ± 11.0) in the probiotics group and 25 patients (male/female 6/19, age 43.2 ± 12.2) in the control group were recruited (Table 1). Baseline median scores of GSRS in the probiotics and control groups were 4.4 ± 2.7 and 4.2 ± 1.9 (*P* = 0.767), weight 66.0 ± 12.5 and 64.8 ± 9.5 (*P* = 0.715), and positive rate of SIBO 60.0% and 52.0% (*P* = 0.254), respectively. 4 weeks later, median scores of GSRS were 1.4 ± 1.4 and 3.6 ± 1.6 (*P* < 0.001, Figure 2), weight 64.9 ± 12.5 and 65.0 ± 9.5 (*P* = 0.990), and positive rate of SIBO 28.0% and 56.0% (*P* < 0.001), respectively.

### 3.2. Fecal Gut Microbiota Composition

Baseline median relative abundances of the gut microbiota in the probiotics and control groups at the phylum level were 2.53% and 1.77% Actinobacteria (*P* = 0.367), 39.88% and 42.46% Bacteroidetes (*P* = 0.992), 51.29% and 45.47% Firmicutes (*P* = 0.377), 0.02% and 0.03% Fusobacteria (*P* = 0.705), 3.18% and 4.25% Proteobacteria (*P* = 0.118), respectively. 4 weeks later, abundances are 1.01% and 5.03% Actinobacteria (*P* < 0.001), 43.80% and 35.17% Bacteroidetes (*P* = 0.008), 42.25% and 48.28% Firmicutes (*P* = 0.240), 0.02% and 0.01% Fusobacteria (*P* = 0.034), and 4.40% and 4.54% Proteobacteria (*P* = 0.180), respectively (Table 2).

Baseline ratios of the gut microbiota in the probiotics and control groups at the phylum level were 1.44 ± 1.10 and 1.38 ± 1.20 Firmicutes to Bacteroidetes ratio (*P* = 0.848) and 0.07 and 0.04 Actinobacteria to Bacteroidetes ratio (*P* = 0.443), respectively. 4 weeks later, we have 1.07 ± 0.51 and 1.60 ± 0.90 Firmicutes to Bacteroidetes ratio (*P* = 0.015) and 0.02 and 0.15 Actinobacteria to Bacteroidetes ratio (*P* < 0.001), respectively (Tables 1 and 2).

**Figure 3:**
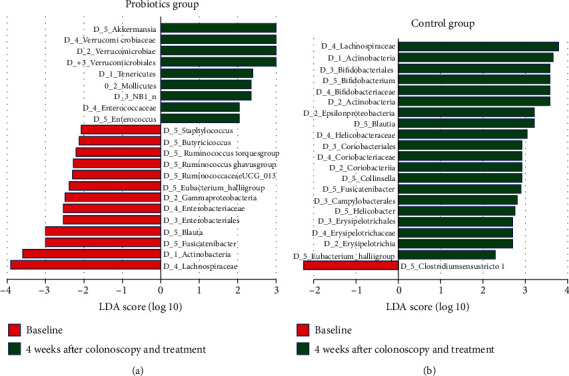
Differences in the composition of the gut microbiota between the baseline and 4 weeks after colonoscopy and treatment groups. Gut bacteria with linear discriminant analysis score > 2.0 between the baseline and 4 weeks after colonoscopy and treatment in the probiotics group (a) and control group (b) at the levels of class, order, family, and genus.

There was no significant difference on the baseline relative abundance at the genus level between the two groups. 4 weeks later, median relative abundances of the gut microbiota in the probiotics and control groups at the genus level were 0.76% and 3.29% *Bifidobacterium* (*P* = 0.011), 0.13% and 0.89% *Cillinsella* (*P* < 0.001), 0.03% and 0.01% *Enterococcus* (*P* = 0.004), 0.18% and 0.36% *Lachnospiraceae (P* = 0.047), 0.10% and 0.16% *Ruminococcus torques group* (*P* = 0.017), 1.31% and 2.44% *Blautia* (*P* = 0.001), 0.08% and 0.16% *Ruminococcaceae UCG0134* (*P* = 0.045), 0.83% and 2.02% *Fusicatenibacter* (*P* = 0.015), and 0.13% and 0.43% Eubacterium hallii group (*P* < 0.001), respectively (Table 3). Gut bacteria with linear discriminant analysis score > 2.0 between the baseline and 4 weeks after colonoscopy and treatment in the probiotics and control groups at the levels of class, order, family, and genus were showed in Figure 3.

**Figure 4:**
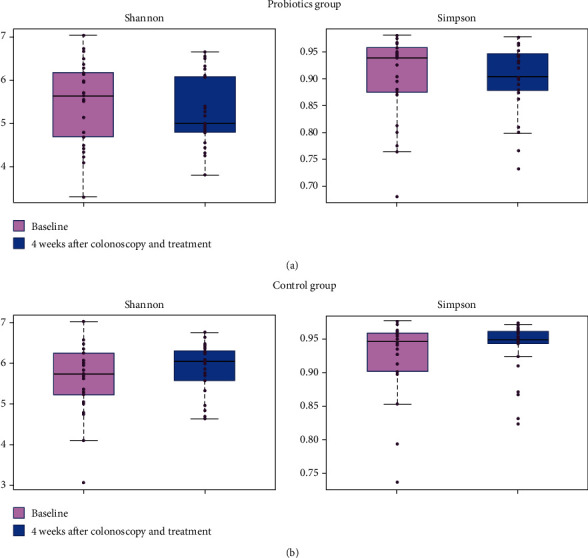
Box plot of the Shannon index and Simpson index in the probiotics group (a) and control group (b). Shannon index and Simpson index, indicating an *α*-diversity, were significantly lower in the 4 weeks after colonoscopy and treatment than those in the baseline of probiotics group. There was no significant difference in the control group.

**Figure 5:**
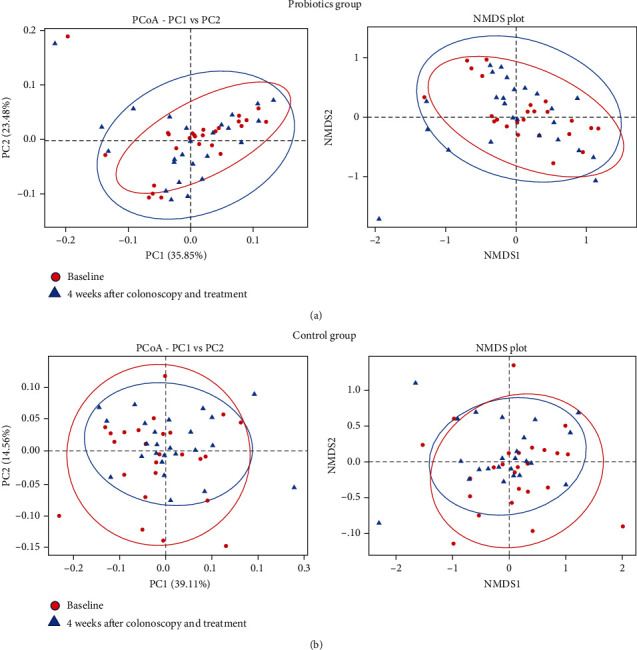
Differences in microbiota composition (*β*-diversity) in the probiotics group (a) and control group (b). Principal coordinate analysis (PCoA) and nonmetric multidimensional scaling (NMDS) analysis between the baseline and 4 weeks after colonoscopy and treatment. The *β*-diversity 4 weeks after colonoscopy and treatment of the probiotics group were more divergent than those in the baseline. However, the *β*-diversity 4 weeks after colonoscopy and treatment of the control group were less divergent than those in the baseline. PC1: principal coordinate 1; PC2: principal coordinate 2.

Shannon index and Simpson index, indicating an *α*-diversity, were significantly lower in the 4 weeks after colonoscopy and treatment than those in the baseline of probiotics group (Figure 4). However, there was no significant difference in the control group. Principal coordinate analysis (PCoA) and nonmetric multidimensional scaling (NMDS) analysis, indicating an *β*-diversity, were more divergent in the 4 weeks after colonoscopy and treatment of the probiotics group than those in the baseline (Figure 5). However, the *β*-diversity in the 4 weeks after colonoscopy and treatment of the control group were less divergent than those in the baseline.

### 3.3. Factors Affecting Fecal Microbiota Composition, GSRS Score, and Decontaminating SIBO

We found age, gender, baseline BMI, and obesity had no significant influence on the changes of fecal microbiota, GSRS score, and SIBO in our study (*P* all > 0.05, Table 4). However, probiotics treatment had a significant influence on the increasing or reducing of the abundances of certain gut bacteria, reducing GSRS score, and decontaminating SIBO.

## 4. Discussion

Probiotics are live microorganisms that play an essential role in human health and disease. The potential mechanisms of probiotics include changing the gut microbiota, interfering with pathogenic bacteria by competitive adherence to the mucosa and competitive nutrition, improving mucosal barrier function, and regulating immune system to convey an advantage to the host [12–14]. Specific probiotic supplementation can relieve lower gastrointestinal symptoms in IBS, prevent diarrhea associated with antibiotics and *Helicobacter pylori* eradication therapy [8, 15], and has an overall insignificant effect on mood and alleviate depressive symptoms [16]. Live combined *Bacillus subtilis* and *Enterococcus faecium* has been prescribed to alleviate symptoms associated with chronic diarrhea and IBS, coadjuvant therapy to improve gastrointestinal symptoms and clinical remission of ulcerative colitis, and to improve *Helicobacter pylori* eradication success rate with conventional triple therapy [17–19], for which can ameliorate gut dysbiosis and inflammation by balancing beneficial and harmful bacteria and associated anti- and proinflammatory agents, thereby aiding gut mucosal repair [20–22]. In our study, we found 4 weeks of live combined *Bacillus subtilis* and *Enterococcus faecium* supplementation after the bowel preparation and colonoscopy can significantly alleviate patient's gastrointestinal symptoms, reduce patients' body weight, and decontaminate SIBO. It is consistent with other studies that specific probiotic supplementation can help reduce abdominal pain, distension, and improve bowel habit [15] and probiotic supplementation can result in a significant reduction in body weight [23].

SIBO can be detected in 4–84% in IBS patient populations by hydrogen breath tests [24], and the clinical manifestations vary widely, from mild gastrointestinal symptoms such as flatulence and bloating to more serious complications including profound weight loss and micronutrient deficiencies, which may result from reducing gastric acid secretion, intestinal dysmotility, ileocecal valve dysfunction, and abnormal immunomodulation [10]. For this reason, several gastrointestinal conditions have been associated with SIBO including inflammatory bowel disease (IBD), IBS, and gastroparesis [10]. Other studies reported increased intestinal gas on abdominal radiograph in IBS, particularly in the small intestine, which supported the relations between SIBO and symptoms such as bloating and flatulence in IBS patients [25, 26]. And at present, glucose and lactulose hydrogen breath tests as noninvasive means are clinically widely performed in the diagnosis of SIBO. In our study, 56% (28/50) patients with gastrointestinal symptom of abdominal pain, abdominal distension, and changing bowel habit suffered SIBO, and after the bowel preparation and 4 weeks of live combined *Bacillus subtilis* and *Enterococcus faecium* supplementation, SIBO positive rate in the probiotics group was significantly reduced, while the control group did not change. Therefore, decontaminated SIBO may lead to alleviating gastrointestinal symptom. Consistent with the meta-analysis result that probiotic supplementation could effectively decontaminate SIBO and relieve abdominal pain [27], and probiotics were not associated with adverse events [28]. In addition to probiotic supplementation, antibiotics are also commonly used to eradicate SIBO. Rifaximin, a nonsystemic antibiotic and poorly absorbed antibiotic with a broad spectrum of antibacterial activity, has been largely used to treat SIBO over the past decades. The improvement or resolution of symptoms in patients with eradicated SIBO was found to be 67.7%, and the overall rate of adverse events was 4.6%. But, well-designed RCTs are needed to substantiate these findings and to establish the optimal regimen [28, 29]. A recent prospective study demonstrated that superior clinical efficacy of four probiotics (Saccharomyces boulardii, Bifidobacterium lactis, Lactobacillus acidophilus, and Lactobacillus plantarum) in patients with IBS and SIBO [30]. However, in a randomized trial that enrolled patients treated with Saccharomyces boulardii and placebo, an overall improvement of the quality of life was detected in the Saccharomyces boulardii group. But, the *Saccharomyces boulardii* group did not show superior reducing individual symptoms in patients with diarrhea-predominant IBS or mixed-type IBS [31].

Our result of the fecal gut microbiota showed that there was an increasing trend in the relative abundance of *Actinobacteria*, but no significant difference on baseline and 4 weeks later relative abundances at the phylum level between the SIBO-positive and SIBO- SIBO-negative groups, which was consistent with Yang et al.'s [24] study states that no significant difference in the composition of fecal microbiota between SIBO-positive and SIBO-negative diarrhea-predominant IBS patients.

FBD patients suffered an increase in the Firmicutes to Bacteroidetes ratio [32, 33], and an increase in the Firmicutes to Bacteroidetes ratio may pose a potential risk to patients' health [34]. In our study, comparing with the control group, the Firmicutes to Bacteroidetes ratio (a reducing trend of Firmicutes abundance and a significant increase of Bacteroidetes abundance at the level of phylum) in the probiotics group significantly reduced after 4 weeks of live combined *Bacillus subtilis* and *Enterococcus faecium* supplementation, with significantly reducing abundance of *Ruminococcus*, *Fusicatenibacter*, and *Eubacterium hallii group* (Firmicutes) at the level of genus, which might help to improve the FBD patients' health. Bacteroidetes play a vital role in degrading complex polysaccharides of cellulose, pectin, and xylan, which can help people absorb more energy from the diet, and butyrate produced by Bacteroidetes plays an important role in maintaining the intestinal health of the host, exerting immunity, and antitumor effect [34].

An adequate bowel preparation can ensure a clear view during colonoscopy and is essential for a successful colonoscopy, and PEG is one of the most worldwide used drugs for the bowel preparation [35]. A study showed that bowel preparation with PEG has a long-lasting effect on the gut microbiota composition and homeostasis in normal individuals, and a significant decrease in Firmicutes abundance and an increase in Proteobacteria abundance immediately at the phylum level after the bowel preparation, and 1 month after the bowel preparation, the abundance of Firmicutes can increase, while the abundance of Proteobacteria decreased [6]. Others studies showed that the gut microbiota composition was significantly reduced immediately after the bowel preparation, but recovered 14 days after the bowel preparation [36, 37]. And previously asymptomatic people may present mild bloating (25%) and abdominal pain (11%) within 30 days after colonoscopy [38]. So, the changing of the gut microbiota after the bowel preparation may cause gastrointestinal symptoms, and the gut microbiota can present reconstruction after the bowel preparation, and probiotic supplementation after the bowel preparation may alter the reconstruction of gut microbiota and may alleviate patient's gastrointestinal symptom. In our study, we found the abundance of Actinobacteria and Actinobacteria to Bacteroidetes ratio increased 4 weeks after the bowel preparation in the control group, and patients' gastrointestinal symptom mildly alleviated. While in the probiotics group, the abundance of Actinobacteria and Actinobacteria to Bacteroidetes ratio reduced and patients' gastrointestinal symptom significantly alleviated, and at the genus level the abundance of *Enterococcus* increased for the supplementation of live *Enterococcus faecium*. Therefore, 4 weeks of live combined *Bacillus subtilis* and *Enterococcus faecium* supplementation can alter the reconstruction of the gut microbiota after the bowel preparation. The difference between our results and the other studies may be due to our patients undergoing afternoon colonoscopy with a same-day bowel preparation different from the split dosing, and in the different selected population in our study, we select the patients with gastrointestinal symptoms, instead of asymptomatic population, which may have different gut microbiota, and symptomatic population is our advantage.

This study had some limitations, such as small sample size and single center research, but we explained the clinically observed phenomena of alleviating the gastrointestinal symptoms of FBDs after the bowel preparation, colonoscopy, and following treatment of live combined *Bacillus subtilis* and *Enterococcus faecium* through a prospectively randomized controlled study, and we will further expand our sample size and perform multicenter research in future study. In summary, live combined *Bacillus subtilis* and *Enterococcus faecium* supplementation after the bowel preparation can alter the gut microbiota, decontaminate SIBO, and alleviate the gastrointestinal symptoms of FBDs.

## Figures and Tables

**Table 1:** Participant data (*x* ± SD, *n*, %).

Items	Probiotics group	Control group	Independent sample *t*-test or chi-square test
	*n* = 25	*n* = 25	
Baseline			
Age (mean ± SD, years)	40.6 ± 11.0	43.2 ± 12.2	*t* = 0.802, *P* = 0.427
Male/female (*n*)	9/16	6/19	*χ* ^2^ = 0.857, *P* = 0.355
GSRS (median IQR)	4.4 ± 2.7	4.2 ± 1.9	*t* = 0.298, *P* = 0.767
BMI (kg/m^2^)	24.1 ± 3.7	24.2 ± 2.7	*t* = 0.904, *P* = 0.925
Weight (kg)	66.0 ± 12.5	64.8 ± 9.5	*t* = 0.368, *P* = 0.715
Positive rate of SIBO (%)	60	52	*χ* ^2^ = 1.299*P* = 0.254
OTU	546.0 ± 73.6	535.4 ± 84.3	*t* = 0.474, *P* = 0.638
Shannon index	5.48 ± 0.97	5.60 ± 0.86	*t* = 0.456, *P* = 0.650
Simpson index	0.90 ± 0.07	0.92 ± 0.06	*t* = 1.089, *P* = 0.282
4 weeks after colonoscopy and treatment			
GSRS (median IQR)	1.4 ± 1.4^a^	3.6 ± 1.6^b^	*t* = 5.244, *P* < 0.001
BMI (kg/m^2^)	23.4 ± 3.5^a^	24.2 ± 2.8	*t* = 0.892, *P* = 0.377
Weight (kg)	64.9 ± 12.5^a^	65.0 ± 9.5	*t* = 0.013, *P* = 0.990
Positive rate of SIBO (%)	28^a^	56	*χ* ^2^ = 16.092, *P* < 0.001
OTU	524.8 ± 72.7	551.6 ± 78.1	*t* = 1.255, *P* = 0.216
Shannon index	5.23 ± 0.84	5.86 ± 0.59	*t* = 3.059, *P* = 0.004
Simpson index	0.90 ± 0.06	0.93 ± 0.04	*t* = 2.219, *P* = 0.031

GSRS: gastrointestinal symptom rating scale; BMI: body mass index; SIBO: small intestinal bacteria overgrowth; SD: standard deviation; IQR: interquartile range; OTU: operational taxonomic unit. ^a^Paired values of one month after colonoscopy vs. baseline in the probiotics group (*P* < 0.05, paired sample *t*-test or Wilcoxon test). ^b^Values of one month after colonoscopy vs. baseline in the control group (*P* < 0.05, paired sample *t*-test or Wilcoxon test).

**Table 2:** Composition of the gut microbiota at the phylum level (median (IQR)).

Items	Probiotics group	Control group	Independent sample *t*-test or Mann–Whitney test
	*n* = 25	*n* = 25	
Baseline			
Actinobacteria (%)	2.53 (0.94–8.13)	1.77 (0.50–2.54)	*Z* = −0.902, *P* = 0.367
Bacteroidetes (%)	39.88 (30.36–47.94)	42.46 (31.77–46.15)	*Z* = −0.010, *P* = 0.992
Firmicutes (%)	51.29 (37.49–55.67)	45.47 (36.21–53.61)	*Z* = −0.883, *P* = 0.377
Fusobacteria (%)	0.02 (0.01–0.05)	0.03 (0.01–0.06)	*Z* = −0.378, *P* = 0.705
Proteobacteria (%)	3.18 (2.40–5.47)	4.25 (3.34–6.60)	*Z* = −1.562, *P* = 0.118
Others (%)	0 (0–0.01)	0 (0–0.03)	*Z* = −0.802, *P* = 0.423
Firmicutes to Bacteroidetes ratio	1.44 ± 1.10	1.38 ± 1.20	*t* = 0.193, *P* = 0.848
Actinobacteria to Bacteroidetes ratio	0.07 (0.02–0.22)	0.04 (0.02–0.07)	*Z* = −0.766, *P* = 0.443
4 weeks after colonoscopy and treatment			
Actinobacteria (%)	1.01 (0.50–2.54)^a^	5.03 (1.55–10.06)^b^	*Z* = −3.619, *P* < 0.001
Bacteroidetes (%)	43.80 (34.72–53.34)	35.17 (26.70–41.67)	*Z* = −2.668, *P* = 0.008
Firmicutes (%)	42.25 (34.96–53.81)	48.28 (39.01–55.21)	*Z* = −1.174, *P* = 0.240
Fusobacteria (%)	0.02 (0.01–0.08)^a^	0.01 (0–0.03)^b^	*Z* = −2.125, *P* = 0.034
Proteobacteria (%)	4.40 (3.27–6.92)^a^	4.54 (2.44–6.29)	*Z* = −0.184, *P* = 0.854
Others (%)	0 (0–0.01)	0.01 (0–0.06)	*Z* = −1.339, *P* = 0.180
Firmicutes to Bacteroidetes ratio	1.07 ± 0.51	1.60 ± 0.90	*t* = 2.522, *P* = 0.015
Actinobacteria to Bacteroidetes ratio	0.02 (0.01–0.02)^a^	0.15 (0.04–0.38)^b^	*Z* = −3.696, *P* < 0.001

IQR: interquartile range. ^a^Paired values of one month after colonoscopy vs. baseline in the probiotics group (*P* < 0.05, Wilcoxon test). ^b^Values of one month after colonoscopy vs. baseline in the control group (*P* < 0.05, Wilcoxon test).

**Table 3:** Composition of the gut microbiota at the genus level (median (IQR), %).

Items	Probiotics group	Control group	Mann–Whitney test
	*n* = 25	*n* = 25	
Baseline			
Bifidobacterium	2.59 (0.41–7.51)	0.80 (0.25–2.40)	*Z* = −1.504, *P* = 0.133
Collinsella	0.29 (0.01–1.04)	0.47 (0.18–1.08)	*Z* = −0.747, *P* = 0.445
Enterococcus	0.01 (0–0.02)	0.01 (0–0.01)	*Z* = −1.113, *P* = 0.266
Lachnospiraceae ND3007	0.20 (0.15–0.35)	0.29 (0.11–0.42)	*Z* = −0.281, *P* = 0.778
Staphylococcus	0 (0–0.003)	0 (0–0.002)	*Z* = −1.184, *P* = 0.237
Ruminococcus torques group	0.25 (0.13–0.40)	0.15 (0.08–0.35)	*Z* = −1.64, *P* = 0.101
Blautia	2.57 (1.57–3.90)	1.77 (1.24–2.47)	*Z* = −1.562, *P* = 0.118
Ruminococcaceae UCG013	0.17 (0.05–0.33)	0.15 (0.02–0.31)	*Z* = −0.689, *P* = 0.491
Akkermansia	0.01 (0–0.02)	0.02 (0–0.64)	*Z* = −1.602, *P* = 0.109
Fusicatenibacter	1.78 (1.04-2.96)	1.41 (0.90-2.22)	*Z* = −0.883, *P* = 0.377
Eubacterium hallii group	0.28 (0.22–0.57)	0.26 (0.21–0.41)	*Z* = −0.786, *P* = 0.432
Butyricicoccus	0.18 (0.10–0.27)	0.12 (0.05–0.28)	*Z* = −1.348, *P* = 0.177
4 weeks after colonoscopy and treatment			
Bifidobacterium	0.76 (0.30–2.28)^a^	3.29 (1.14–8.72)^b^	*Z* = −2.551, *P* = 0.011
Collinsella	0.13 (0.01–0.44)^a^	0.89 (0.30–2.18)^b^	*Z* = −3.968, *P* < 0.001
Enterococcus	0.03 (0.01–0.12)^a^	0.01 (0–0.04)^b^	*Z* = −2.844, *P* = 0.004
Lachnospiraceae ND3007	0.18 (0.05–0.35)	0.36 (0.22–0.53)	*Z* = −1.989, *P*=0.047
Staphylococcus	0 (0–0)^a^	0 (0–0.002)	*Z* = −1.043, *P* = 0.297
Ruminococcus torques group	0.10 (0.04–0.15)^a^	0.16 (0.11–0.33)	*Z* = −2.396, *P* = 0.017
Blautia	1.31 (0.65–1.68)^a^	2.44 (1.36–4.78)^b^	*Z* = −3.211, *P* = 0.001
Ruminococcaceae UCG013	0.08 (0.02–0.13)^a^	0.16 (0.03–0.54)	*Z* = −2.008, *P* = 0.045
Akkermansia	0.06 (0.05–0.08)^a^	0.03 (0.01–0.49)	*Z* = −1.640, *P* = 0.101
Fusicatenibacter	0.83 (0.48–1.72)^a^	2.02 (0.78–2.83)^b^	*Z* = −2.435, *P*=0.015
Eubacterium hallii group	0.13 (0.05–0.25)^a^	0.43 (0.33–0.64)^b^	*Z* = −4.628, *P* < 0.001
Butyricicoccus	0.08 (0.04–0.16)^a^	0.18 (0.07–0.29)	*Z* = −2.377, *P* = 0.017

IQR: interquartile range. ^a^Paired values of one month after colonoscopy vs. baseline in the probiotics group (*P* < 0.05, Wilcoxon test). ^b^Values of one month after colonoscopy vs. baseline in the control group (*P* < 0.05, Wilcoxon test).

**Table 4:** Results of linear regression analysis (*P* values).

Changed of items	Probiotics treatment	Baseline BMI	Baseline obesity	Age	Gender
OTU	0.557	0.697	0.539	0.935	0.386
Shannon index	0.005	0.189	0.922	0.281	0.576
Simpson index	0.204	0.401	0.363	0.458	0.831
Actinobacteria	<0.001	0.498	0.957	0.194	0.189
Bacteroidetes	0.036	0.625	0.407	0.382	0.788
Firmicutes	0.632	0.823	0.593	0.302	0.059
Fusobacteria	0.042	0.281	0.865	0.737	0.561
Proteobacteria	0.469	0.263	0.358	0.265	0.450
Other	0.113	0.313	0.696	0.267	0.972
GSRS	0.001	0.855	0.842	0.501	0.851
SIBO	0.017	0.243	0.287	0.531	0.670

BMI: body mass index; GSRS: gastrointestinal symptom rating scale; SIBO: small intestinal bacteria overgrowth; OTU: operational taxonomic unit.

## Data Availability

Relevant raw data from this study are available upon request. Please contact the corresponding author.
